# Embryonic disc formation following post-hatching bovine embryo development *in vitro*

**DOI:** 10.1530/REP-20-0243

**Published:** 2020-07-17

**Authors:** Priscila Ramos-Ibeas, Ismael Lamas-Toranzo, Álvaro Martínez-Moro, Celia de Frutos, Alejandra C Quiroga, Esther Zurita, Pablo Bermejo-Álvarez

**Affiliations:** 1 Departamento de Reproducción Animal, INIA, Madrid, Spain

## Abstract

Failures during conceptus elongation are a major cause of pregnancy losses in ungulates, exerting a relevant economic impact on farming. The developmental events occurring during this period are poorly understood, mainly because this process cannot be recapitulated in vitro. Previous studies have established an in vitro post-hatching development (PHD) system that supports bovine embryo development beyond the blastocyst stage, based on agarose gel tunnels and serum- and glucose-enriched medium. Unfortunately, under this system embryonic disc formation is not achieved and embryos show notorious signs of apoptosis and necrosis. The objective of this study has been to develop an in vitro system able to support embryonic disc formation. We first compared post-hatching development inside agarose tunnels or free-floating over an agarose-coated dish in serum- and glucose-enriched medium (PHD medium). Culture inside agarose tunnels shaped embryo morphology by physical constriction, but it restricted embryo growth and did not provide any significant advantage in terms of development of hypoblast and epiblast lineages. In contrast to PHD medium, a chemically defined and enriched medium (N2B27) supported complete hypoblast migration and epiblast survival in vitro, even in the absence of agarose coating. Cells expressing the pluripotency marker SOX2 were observed in ~56% of the embryos and ~25% developed embryonic disc-like structures formed by SOX2+ cells. In summary, here we provide a culture system that supports trophectoderm proliferation, hypoblast migration and epiblast survival after the blastocyst stage.

## Introduction

Understanding ungulate embryo development during the first 2 weeks after fertilization is critical to improve reproductive efficiency in livestock, since the greatest gestational losses occur within this period, leading to an important economic impact on farming (Ayalon 1978, Diskin & Morris 2008, Berg *et al.* 2010). Focussing on cattle, around one-third of the viable blastocysts fail to achieve conceptus elongation in vivo (Santos *et al.* 2004). Conceptus elongation entails several critical developmental processes, including the development of extraembryonic membranes and embryonic disc (van Leeuwen *et al.* 2015). By day 9 of development, two different lineages have emerged from the inner cell mass (ICM): the epiblast and the hypoblast (Maddox-Hyttel *et al.* 2003). The hypoblast will migrate to cover the entire blastocyst, lying beneath both the epiblast and the trophectoderm, whereas the epiblast will form the embryonic disc. Towards the end of the second week of pregnancy, the extraembryonic membranes experience a massive growth by which the embryo elongates, the epiblast forms a one- or two-layered epithelium and the trophectoderm covering the epiblast (Rauber’s layer) disappears (van Leeuwen *et al.* 2015). Unfortunately, bovine in vitro embryo culture endpoint is restricted to hatched blastocysts on day 8 or 9 of development, impairing the investigation of more developmentally advanced stages. Alternatively, day 7 blastocysts can be transferred to surrogate mothers and elongated embryos can be recovered on days 14–16 of development (van Leeuwen *et al.* 2015), although with an elevated effort and economical cost.

Previous studies have established different in vitro culture systems to support development beyond the blastocyst stage in bovine embryos (Brandao *et al.* 2004, Vajta *et al.* 2004, Alexopoulos *et al.* 2005). The most advanced method is a post-hatching development (PHD) system, where embryos are cultured inside agarose gel tunnels filled with serum- and glucose-enriched medium (PHD medium) until day 15 or 16. The embryos developed in this system showed a defined mass of epiblast-derived cells, Rauber’s layer and some degree of proliferation of hypoblast cells inside the epiblast and trophoblast (Brandao *et al.* 2004, Vajta *et al.* 2004, Machado *et al.* 2013). However, it remains unclear whether the hypoblast covers entirely the inner surface of the embryo, and notorious signs of apoptosis and necrosis could be noticed, especially in epiblast-derived cells.

PHD system relies on two main differential features compared to conventional pre-hatching in vitro systems: the use of agarose tunnels as a culture substrate and a higher concentration of glucose and serum. Agarose substrate prevents the attachment of the embryo to the bottom of the culture dish, extending the window of embryo development in vitro, but the metabolic pathways by which this medium supports embryo development in vitro remain unexplored. Nevertheless, it remains unclear whether embryo culture inside agarose tunnels enhances embryo development and triggers elongation or if these embryos are only shaped to a tubular form due to the restricted space inside the tunnel. Furthermore, alternative culture substrates and enriched media that could support epiblast survival and the formation of the embryonic disc have not been explored. The objective of this study has been to develop an in vitro system to achieve embryonic disc formation during post-hatching bovine embryo development. To this aim, we have evaluated the effects of different culture substrates (agarose tunnels, agarose layer or no agarose) and media (PHD or N2B27 media) on bovine post-hatching embryo development in vitro.

## Materials and methods

### *In vitro* production of bovine blastocysts

The techniques for in vitro embryo production have been described in detail previously (Bermejo-Alvarez *et al.* 2011, Lamas-Toranzo *et al.* 2018). Briefly, immature cumulus-oocyte complexes (COCs) were obtained by aspirating follicles (2–8 mm) from bovine ovaries collected at a local slaughterhouse. COCs were matured for 24 h in TCM-199 supplemented with 10% (v/v) fetal calf serum (FCS) and 10 ng/mL EGF at 39°C under an atmosphere of 5% CO_2_ in air with maximum humidity. For in vitro fertilization (IVF), matured COCs were inseminated with frozen-thawed Bovi-Pure (Nidacon) separated bull sperm at a final concentration of 10^6^ spermatozoa/mL. Gametes were co-incubated in four-well dishes containing 50 COCs and 500 µL of TALP medium per well at 39°C in an atmosphere of 5% CO_2_ and maximum humidity. Semen from the same bull was used for all the experiments to avoid a possible confounding bull effect on developmental rates and to reduce inter-replicate variation. At approximately 20 h post-insemination (hpi), presumptive zygotes were denuded and cultured in groups of ~25 in 25 μL droplets under mineral oil. Culture took place in synthetic oviduct fluid (SOF) (Holm *et al.* 1999) supplemented with 5% FCS at 39°C under an atmosphere of 5% CO_2_, 5% O_2_, and 90% N_2_ with maximum humidity.

*In vivo* embryos were obtained from two Holstein cows following the superovulation protocol described in Supplementary Fig. 1 (see section on supplementary materials given at the end of this article), which involved progesterone (CIDR®, Zoetis), PGF2α analog (cloprostenol, Estrumate®, MSD), GnRH (Dalmarelin®, Fatro Ibérica), and follicle-stimulating hormone and luteinizing hormone (FSH-LH, Pluset®, Calier). Conceptuses were recovered 11.5 days (E11) or 14.5 days (E14) after first insemination by uterine flushing using an 18 mm embryo flushing catheter (Minitüb).

### Post-hatching development system

**Figure 1:**
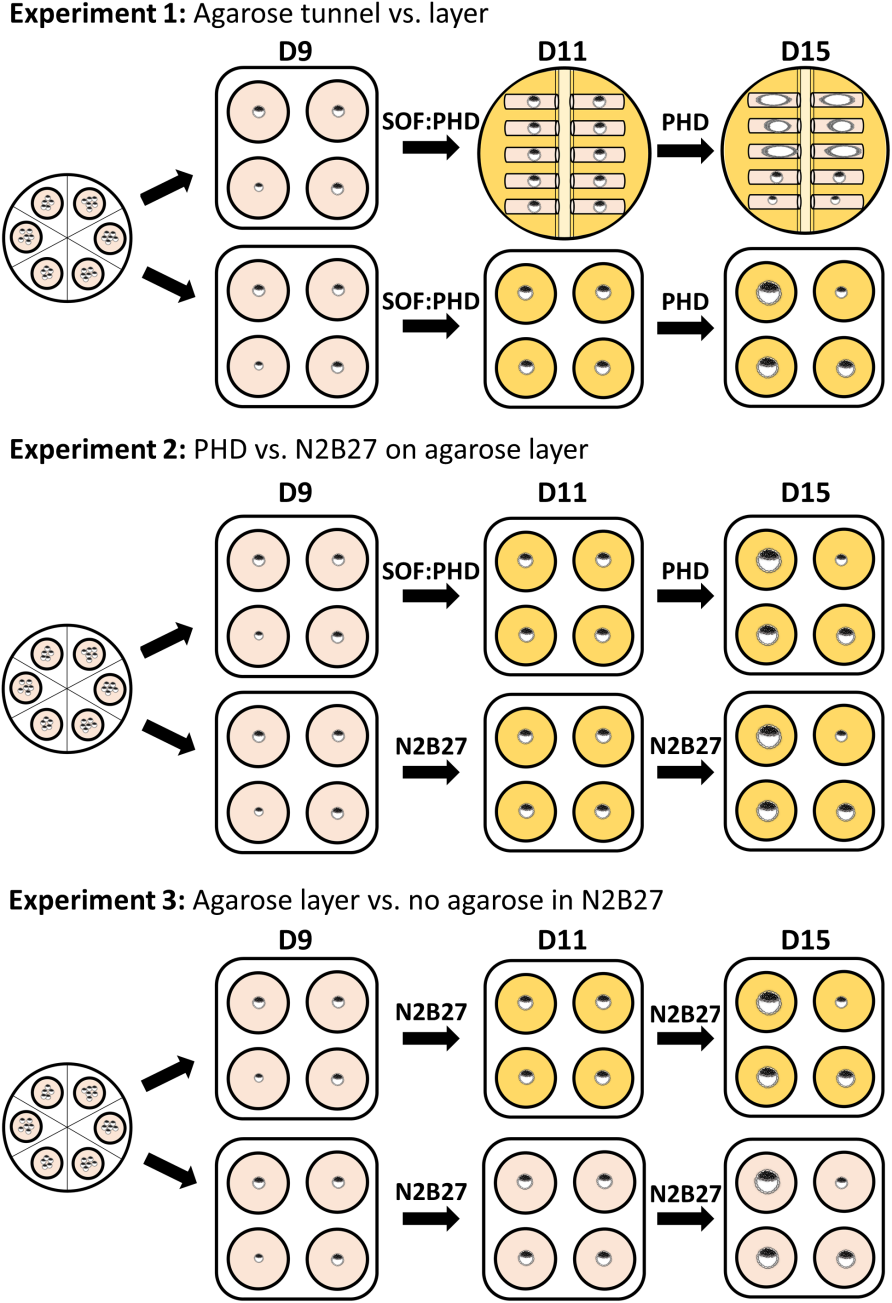
Experimental design. All experiment started from D9 blastocysts produced in vitro in SOF medium. Experiment 1 compared the original PHD system in agarose tunnel (original system, depicted as a yellow dish with tunnels) vs culture on agarose layer (represented as yellow wells) using the same media: SOF:PHD from D9 to D11 (without agarose) and PHD from D11 to D15 (with agarose tunnel or layer). Experiment 2 compared PHD vs N2B27 media using agarose layer as culture support from D11 to D15. Experiment 3 compared agarose layer vs no layer (illustrated as pink wells) using N2B27 medium.

Three independent experiments were carried out to compare different conditions for post-hatching embryo development: (1) agarose tunnels vs agarose layer in PHD medium, (2) PHD medium vs N2B27 medium over agarose layer, and (3) agarose layer vs no layer in N2B27 medium (Fig. 1). Agarose gels were prepared 3 days before use and covered with PHD or N2B27 media (Brandao *et al.* 2004). Four-well dishes were covered with agarose gel for culture on a layer. To prepare agarose tunnels, 1 mm wide glass capillaries (World Precision Instruments) were cut to 60 mm length and one of the open ends was closed by melting the glass over a gas flame. Eight capillaries were oriented in parallel and fixed with tape at the open end. Two of the resulting combs were placed in the opposite direction in a 60 mm Petri dish with the closed ends placed in the bottom (Supplementary Fig. 2A). Gel was prepared by solving 2.4% ultrapure low melting point agarose (Thermo Fisher Scientific) in PBS. The solution was autoclaved, 10% FBS was added once cooled down to 45°C, and then immediately poured into the culture dishes. Dishes were placed in ice bags for rapid solidification of the gel, PHD medium was poured on the gel surface and capillaries were carefully removed. Tunnels were cut to 15 mm length and excess gel was removed. Dishes were kept at 39°C and 5% CO_2_ in water-saturated atmosphere, medium was replaced and the lumen of the tunnels was washed daily with a pulled and curved Pasteur pipette until utilization.

All post-hatching development systems tested took place at 39°C in a water-saturated atmosphere of 5% CO_2_, 5% O_2_, and 90% N_2_. Day 9 (D9) in vitro produced blastocysts were transferred to 1:1 SOF:PHD medium (SOF supplemented with 0.5% glucose and 10% FBS) for PHD groups or to N2B27 medium (1:1 Neurobasal and DMEM/F12 medium supplemented with penicillin/streptomycin, 2 mM glutamine, N2 and B27, Thermo Fisher Scientific) for N2B27 groups. Day 9 to day 11 (D11) culture took place without agarose. On D11, hatched blastocysts showing trophoblast expansion and a defined inner cell mass were transferred to agarose tunnel or layer in PHD medium (experiment 1), to agarose layer in PHD or N2B27 media (experiment 2) or to N2B27 medium with or without agarose layer (experiment 3) (Fig. 1). Pictures were taken to determine the initial embryo size at D11. Embryos remained in culture until day 15 (D15), when pictures were taken to measure embryo length, area and volume. At the end of the culture, those embryos reaching a significant growth were collected for further analyses.

### Embryo measurements

Pictures from the embryos were taken at D11 and D15 on a stereomicroscope (Zeiss Stemi 305). Length and area were measured using Fiji software (Schindelin *et al.* 2012). Elongated embryos were classified as spherical or cylindrical. Volume was calculated using the formula 
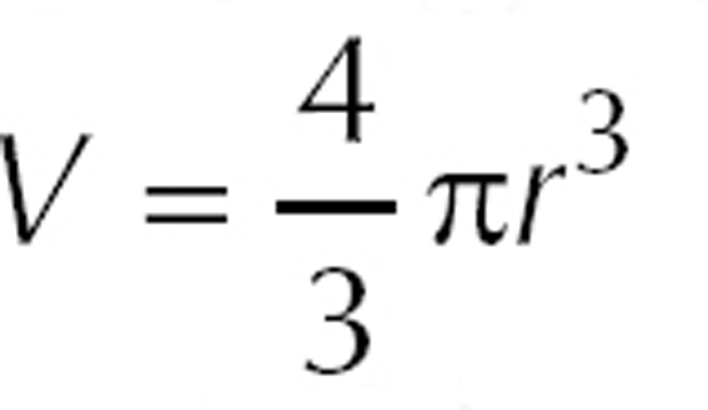
 for spherical embryos. Cylindrical embryos were subdivided into a sphere and a cylinder. Total embryo volume was calculated by adding the volume of a sphere and the volume of a cylinder, calculated with the formula *V* = π*r*^2^*h*. As very few embryos smaller than 0.5 mm in diameter at D11 survived to D15, and D11 initial embryo size could influence D15 embryo dimensions, only D15 embryos developing from D11 embryos larger than 0.5 mm were used for analysis.

### Immunofluorescence

Embryos were fixed in 4% paraformaldehyde (PFA) for 15 min at room temperature (RT), washed in PBS-1% BSA, permeabilized in 1% Triton X-100 in PBS for 15 min at RT and blocked in 10% Donkey Serum-0.02% Tween 20 in PBS for 1 h at RT. Then, embryos were incubated overnight at 4°C with primary antibodies to detect trophectoderm (CDX2, Biogenex MU392A-UC 1:100 dilution), hypoblast (SOX17, R&D AF1924, 1:100 dilution), or epiblast (SOX2, Invitrogen 14-9811-80, 1:100 dilution) cells. After four washes in PBS-1% BSA, embryos were incubated in the appropriate secondary Alexa-conjugated antibodies (Life Technologies) and DAPI for 1 h at RT, followed by four washes in PBS-1% BSA. Finally, embryos were mounted and imaged at a Nikon Eclipse TE 300 fluorescence microscope or at a Leica TCS-SP5 AOBS laser scanning confocal microscope (Leica Microsystems). For tridimensional images, embryos were placed on PBS-1% BSA microdrops made by drawing circles with a PAP pen (Kisker Biotech GmbH) on a coverslide, as previously described (Bermejo-Alvarez *et al.* 2012). Microdrops were covered by an incubation chamber (Sigma Z37,9467) to prevent embryo crushing (Supplementary Fig. 2B). The number of embryos analyzed were 25 in experiment 1 (12 and 13 in tunnel and layer groups, respectively), 25 in experiment 2 (15 and 10 in PHD and N2B27 groups, respectively) and 27 in experiment 3 (11 and 16 in layer and no layer groups, respectively).

### RNA isolation, cDNA synthesis and qPCR

Poly (A) RNA was extracted from five individual whole D15 embryos of each group and five pools of five D9 blastocysts using the Dynabeads mRNA Purification Kit (Life Technologies) following the manufacturer’s instructions with minor modifications (Bermejo-Alvarez *et al.* 2011). Briefly, 100 µL of lysis buffer were added to the sample and incubated at RT for 10 min with gently shaking. Then, 20 µL of beads were added and samples were incubated at RT for 5 min with gentle shaking, allowing beads/mRNA complexes formation. Finally, beads/mRNA complexes were washed twice in washing buffer A and twice in washing buffer B and resuspended in 10 mM Tris–HCl pH 7.5. The amount of mRNA/sample was roughly similar, being around 4 ng. Immediately after extraction, samples were treated with DNAse (Promega) at 37°C for 5 min followed by enzyme denaturalization at 90°C for 5 min, and then the RT reaction was carried out with qScript cDNA Supermix (Quantabiosciences, Gaithersburg, MS, USA) in a total volume of 20 µL. Tubes were first incubated at 25°C for 5 min and then at 42°C for 60 min to allow the RT of RNA, followed by 85°C for 5 min to denature the reverse transcriptase. mRNA transcripts were quantified by real-time quantitative PCR (qPCR). Two replicate PCR experiments were conducted for all genes of interest and qPCR efficiency was tested beforehand; all primers used showed efficiencies above 0.9. PCR was performed by adding a 2-µL aliquot of each sample to the PCR mix (GoTaq qPCR Master Mix, Promega) containing the specific primers. Primer sequences are provided in Supplementary Table 1. The comparative cycle threshold (CT) method was used to quantify expression levels. Fluorescence was acquired in each cycle to determine the threshold cycle. According to the comparative CT method, the CT value was determined by subtracting the endogenous control *H2AFZ* CT value – tested for stability on previous publications (Bermejo-Alvarez *et al.* 2010*b*, 2011) – for each sample from the CT value of each gene in the sample. CT was calculated using the highest sample CT value (i.e. the sample with the lowest target expression) as an arbitrary constant to be subtracted from all other CT sample values. Fold changes in the relative gene expression of the target were determined using the formula 2^–ΔΔCT^ (Schmittgen & Livak 2008).

### Statistical analysis

Data were analyzed using the GraphPad Prism (GraphPad Software) software package and a value of *P* < 0.05 was considered significant. Chi-square test was used to analyze the differences in survival between groups. Differences in length, area and volume between groups were analyzed by Student’s *t*-test when data distribution was normal. When normality test failed, statistical differences were analyzed by Mann–Whitney Rank Sum Test. Embryo size at D11 was not used as a co-factor given that D11 embryos smaller than 0.5 mm were excluded for D15 measurement analysis, as most did not survive to that day. Differences in mRNA expression were analyzed by one-way ANOVA (experiment 1) or *t*-test (experiment 2).

## Results

### Post-hatching development in agarose tunnel or without physical constriction

**Figure 2:**
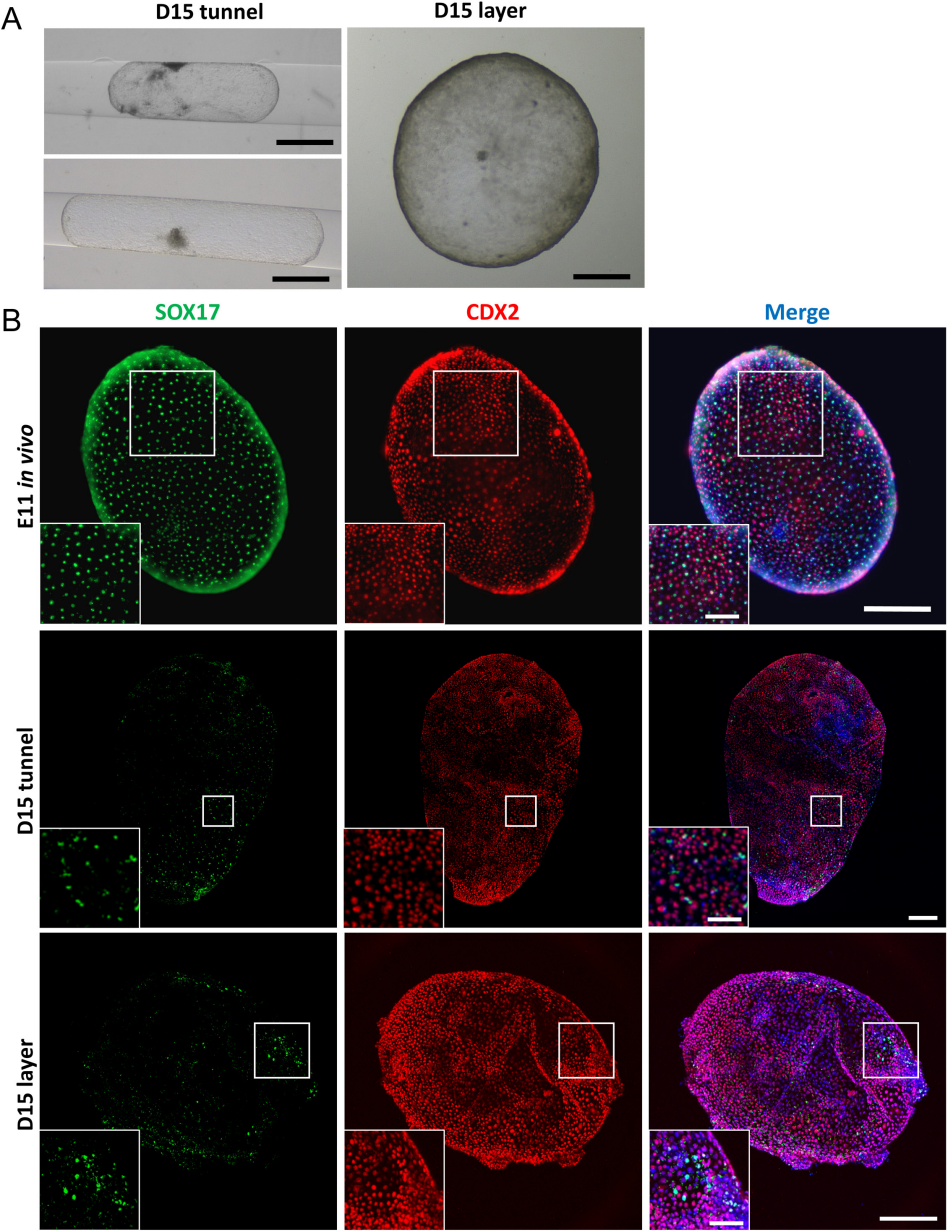
Trophoblast and hypoblast development in agarose tunnel or layer in PHD medium. (A) Representative brightfield stereomicroscopic images of bovine D15 embryos cultured in PHD medium in agarose tunnel or layer. (B) The complete hypoblast migration observed already in embryonic day 11 (E11) in vivo embryos (upper row) contrasts to the incomplete hypoblast layer migration observed in D15 in vitro embryos cultured in PHD medium in agarose tunnel or layer (lower rows). Immunofluorescence staining for SOX17 (hypoblast) and CDX2 (trophoblast); nuclei were counterstained with DAPI (merge). Pictures in the corner are magnifications. Scale bars = 1 mm for A; 300 µm for whole embryo pictures in B; 100 µm for magnifications.

In order to assess the effect of embryo culture inside agarose tunnels on post-hatching development, in vitro produced bovine embryos were cultured in agarose tunnels or free-floating over a layer of agarose, in high serum and glucose medium (PHD medium) according to previously described methods (Brandao *et al.* 2004, Vajta *et al.* 2004). On day 11 of development, embryos were measured and randomly allocated to the two different culture systems (tunnel or layer) until day 15, when embryo survival and growth were assessed. The main factor determining embryo survival was the initial embryo size at D11. When initial embryo diameter was smaller than 0.5 mm, only 1 out of 37 (~3%) and 1 out of 22 (~5%) embryos survived in agarose tunnel and layer, respectively. However, when embryo diameter was 0.5 mm or more, 23 out of 32 (~72%) embryos cultured in agarose tunnel and 19 out of 26 (~73%) embryos cultured over agarose layer survived. No differences were observed in embryo survival between agarose tunnel and layer systems. Although surviving embryos cultured inside tunnels displayed an elongated form distinct to the spherical shape of those cultured over an agarose layer (Fig. 2A), embryo length (i.e. the maximum dimension) was similar between both groups. However, area and volume of surviving embryos were significantly higher in embryos cultured over an agarose layer compared to those cultured inside an agarose tunnel, indicating that culture inside a tunnel restricted embryo growth (Table 1).

**Table 1:** Average length, area and volume of surviving embryos at days 11 and 15 of development.

	*n*	D11 length (mm)	D11 area (mm^2^)	D11 volume (mm^3^)	D15 length (mm)	D15 area (mm^2^)	D15 volume (mm^3^)
Tunnel	23	0.67 ± 0.02	0.35 ± 0.02	0.17 ± 0.02	2.20 ± 0.31	1.92 ± 0.31^a^	1.52 ± 0.24^a^
Layer	19	0.77 ± 0.04	0.46 ± 0.04	0.27 ± 0.04	2.09 ± 0.22	3.98 ± 0.92^b^	7.50 ± 2.33^b^

Data are mean ± s.e.m. Within columns, values followed by different superscript letters differ significantly (*P* < 0.05, Mann–Whitney Rank Sum Test).

**Figure 3:**
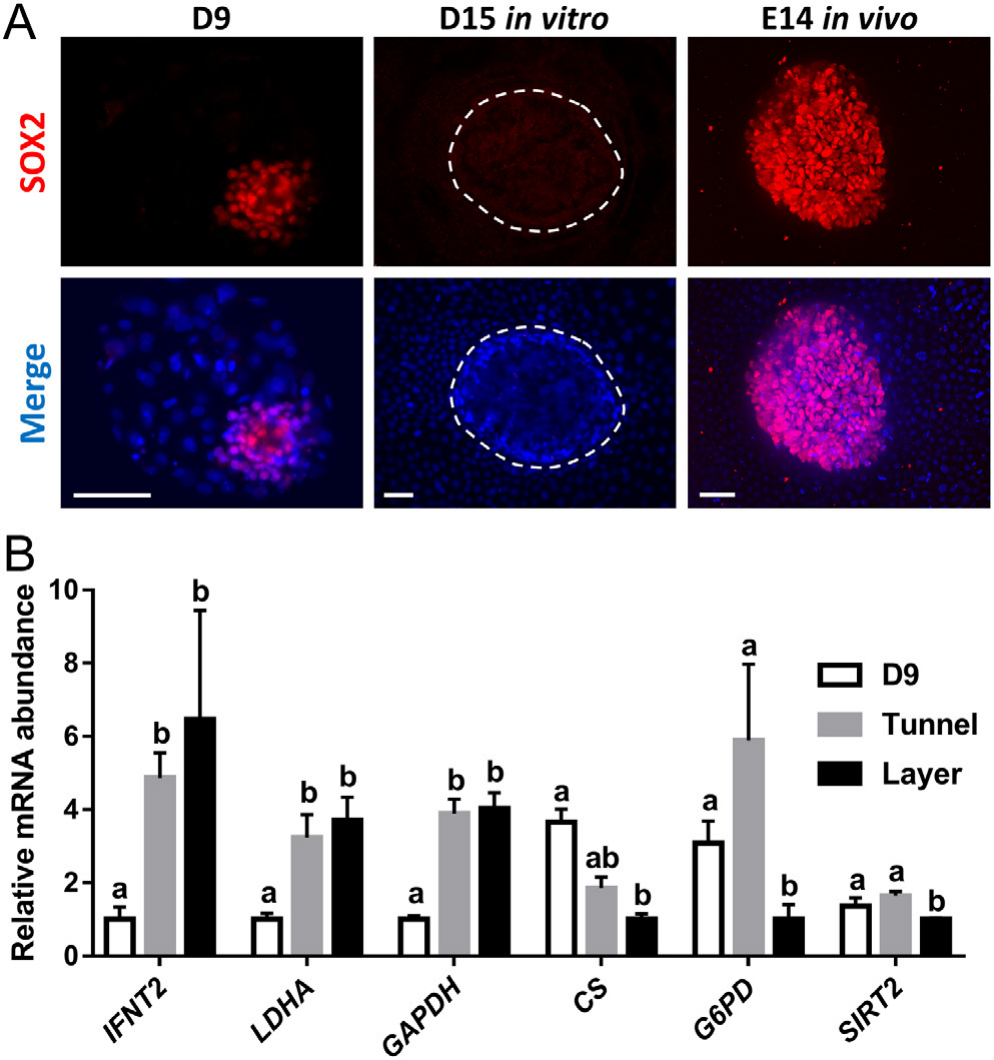
Immunofluorescence detection of epiblast cells and relative mRNA abundance following culture in agarose tunnel or layer in PHD medium. (A) Epiblast development is impaired in D15 embryos developed in PHD medium. Immunofluorescence staining for SOX2 (epiblast) in representative D9 blastocyst (left column), D15 embryo developed in vitro in PHD medium (medium column) and E14 in vivo developed embryonic disc. Nuclei were counterstained with DAPI (merge). Scale bars = 50 µm. (B) Relative mRNA abundance in D9 and D15 embryos cultured in agarose tunnel or layer in PHD medium. Different letters indicate significant differences based on one-way ANOVA (*P* < 0.05).

To assess lineages differentiation, hypoblast cells were detected by immunostaining for SOX17 (Negrón-Pérez *et al.* 2017). A layer of SOX17+ cells was detected inside the CDX2+ trophectoderm in most of the embryos that grew until D15 in both culture conditions. However, in contrast to in vivo derived embryos, hypoblast cells did not cover the entire inner embryo surface (Fig. 2B and Supplementary Fig. 3). The surface of the embryo covered by hypoblast was very variable and there were no significant differences between both conditions (Table 2). Day 9 in vitro-produced embryos, that is, prior to culture in PHD medium, showed clear SOX2+ cells in the ICM. However, although a compact mass of epiblast-derived cells was detected in some developmentally advanced embryos following culture in PHD medium, no SOX2-positive cells were detected in any culture condition (*n* = 12 cultured in tunnel and 13 embryos cultured over agarose layer) and the nuclei of the degenerating masses of epiblast-derived cells appeared disorganized, in contrast to epiblast cells from an in vivo derived embryonic disc (Fig. 3A).

**Table 2:** Hypoblast development in embryos cultured in agarose tunnel or layer at day 15.

	*n*	Embryos with hypoblast cells	Embryo surface covered by hypoblast cells			
			≤24%	25–49%	50–74%	≥75%
Tunnel	12	9 (75%)	4 (33.3%)	2 (16.7%)	2 (16.7%)	1 (8.3%)
Layer	13	10 (76.9%)	3 (23.1%)	2 (15.4%)	2 (15.4%)	3 (23.1%)

No significant differences were found (*P* > 0.05, Fisher exact test).

### Culture in PHD medium triggers interferon Tau production and induces a metabolic switch in the embryo

Transcriptional analysis of *IFNT2* expression, the principal embryonic signal for pregnancy recognition in ruminants (Imakawa *et al.* 1987, Helmer *et al.* 1989), revealed an upregulation in D15 embryos cultured in PHD medium compared to D9 blastocysts irrespective of the culture condition (tunnel or layer), reflecting the proliferation and functional development of the trophectoderm lineage. In order to elucidate a possible metabolic switch during post-hatching embryo culture, the expression of several rate-limiting enzymes involved in anaerobic glycolysis (*GAPDH* and *LDHA* (Granchi *et al.* 2010)), pentose phosphate pathway (*G6PD* and its positive regulator *SIRT2* (Wang *et al.* 2014)) and Kreb’s cycle (citrate synthase, *CS* (Seedorf *et al.* 1986)) were analyzed on D15 and D9 embryos. Irrespective of the culture substrate (i.e. tunnel or layer), the anaerobic glycolysis enzymes *GAPDH* and *LDHA* were upregulated in D15 embryos compared to D9 embryos. However, the rate-limiting enzyme for pentose phosphate pathway *G6PD* and its positive regulator *SIRT2* were downregulated in D15 embryos cultured in agarose layer compared to those cultured in tunnel and D9 embryos. Citrate synthase (*CS*) expression was also reduced in D15 embryos cultured in agarose layer, pointing to a decrease in oxidative phosphorylation (Fig. 3B).

### Post-hatching development in a chemically defined enriched medium

**Figure 4:**
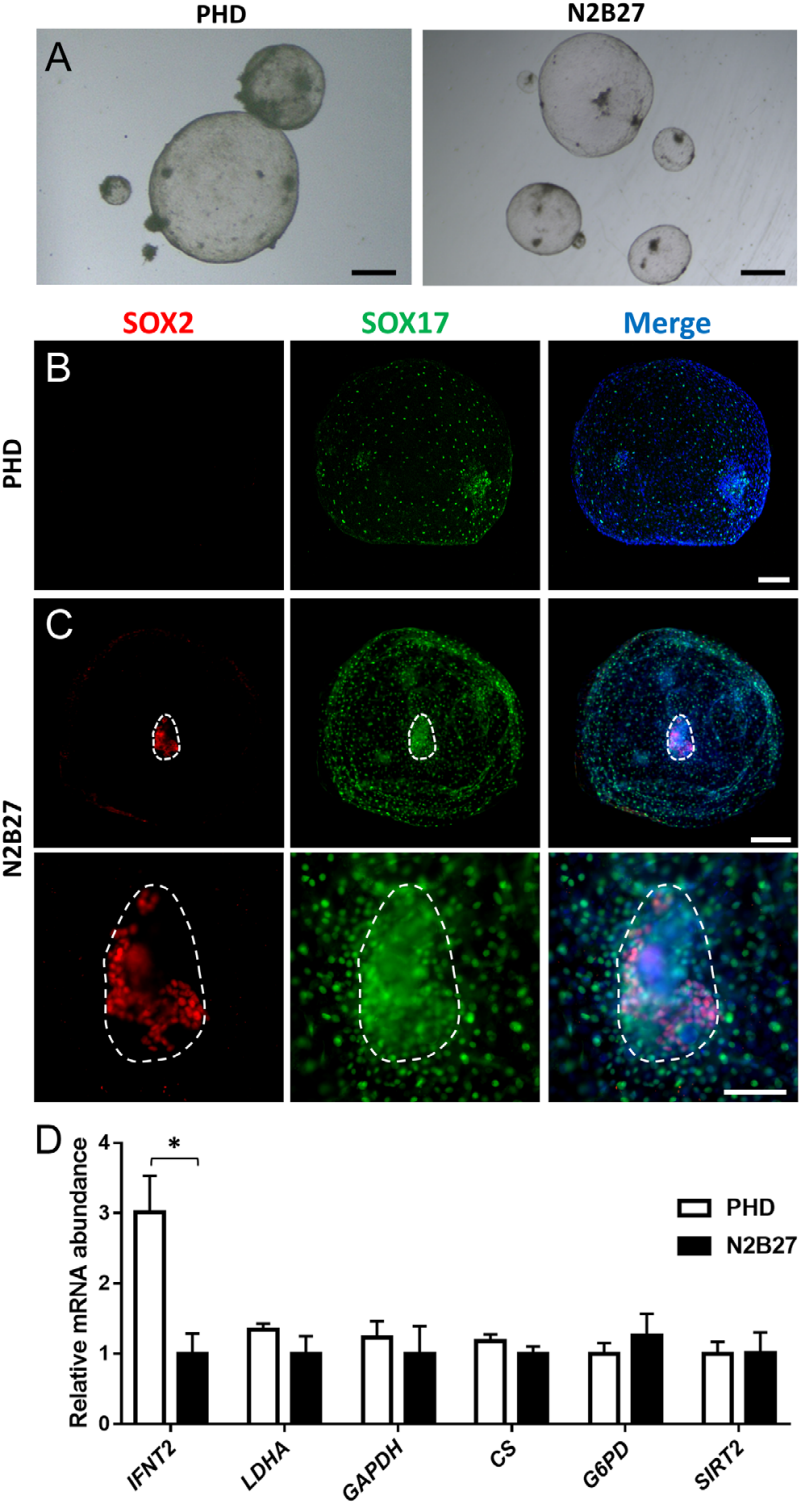
Hypoblast and epiblast development and relative mRNA abundance in embryos developed in PHD or N2B27 media. (A) Representative brightfield stereomicroscopic images of D15 embryos cultured in PHD or N2B27 media over agarose layer. (B) Epiblast and hypoblast development on the only D15 embryo developed in PHD system showing complete hypoblast migration following culture. Notice the reduced density of hypoblast cells compared with C. (C) Epiblast and hypoblast development on a D15 embryo developed in N2B27 medium over agarose layer. Lower row is a magnification of the compact SOX2+ structure resembling an embryonic disc. Immunofluorescence staining for SOX2 (epiblast) and SOX17 (hypoblast); nuclei were counterstained with DAPI (merge). Scale bars = 1 mm for A; 200 µm for B and C upper row; 100 µm for magnification in C (lower row). (D) Relative mRNA abundance in D15 embryos cultured in PHD or N2B27 media. Asterisk indicates significant differences based on *t*-test (*P* < 0.05).

Irrespective of the culture substrate used, hypoblast and epiblast development was compromised in PHD medium: hypoblast migration along the entire inner embryo surface was not accomplished and epiblast development was abolished. These findings suggest that, while PHD medium supports the proliferation of trophectoderm cells, it is unable to sustain embryo development beyond the blastocyst stage. Aiming to provide a better-suited culture medium, we compared post-hatching embryo development in PHD medium vs N2B27, a defined serum-free enriched medium (Supplementary Table 2) suitable for stem cell culture (Ying & Smith 2003). Given that physical constriction was previously proven to be dispensable and even slightly detrimental for embryo development, embryos were cultured on agarose layer in both media. D9 in vitro produced blastocysts were randomly allocated to 1:1 PHD:SOF or to N2B27 medium and 2 days later (D11), embryos were measured and transferred to agarose-coated dishes with PHD or N2B27 medium, respectively. Embryo survival from D11 to D15 was similar in both media. As previously observed, D11 embryo size was the main determinant of embryo survival. Two out of 27 (~7%) and 7 out of 24 (~29%) D11 blastocysts with initial diameter smaller than 0.5 mm survived to D15 in PHD and N2B27 media, respectively. In contrast, 23 out of 35 (~66%) and 15 out of 23 (~65%) D11 embryos larger than 0.5 mm survived in PHD and N2B27 media, respectively. Surviving embryos developed in a spherical shape in both culture media (Fig. 4A), but diameter, area and volume were significantly higher for those embryos cultured in PHD medium (Table 3). As previously noticed (Table 2), hypoblast proliferation was limited in PHD medium: only 1 out of 15 embryos analyzed showed complete hypoblast migration (Fig. 4B). In contrast, all embryos cultured in N2B27 showed complete migration of the hypoblast along the inner embryo surface (*n* = 10, Fig. 4C). Furthermore, while SOX2+ epiblast cells were not observed in any embryo cultured in PHD medium, six out of ten (60%) of the embryos cultured in N2B27 medium showed SOX2+ cells and two of them (20%) showed a compact structure resembling an embryonic disc (Fig. 4C).

**Table 3:** Average length, area and volume of surviving embryos cultured on agarose layer in PHD vs N2B27 media at days 11 and 15 of development.

	*n*	D11 length (mm)	D11 area (mm^2^)	D11 volume (mm^3^)	D15 length (mm)	D15 area (mm^2^)	D15 volume (mm^3^)
PHD	23	0.76 ± 0.07	0.44 ± 0.05	0.25 ± 0.04	2.23 ± 0.16^a^	4.55 ± 1.07^a^	8.84 ± 2.74^a^
N2B27	15	0.65 ± 0.06	0.35 ± 0.03	0.15 ± 0.03	1.6 ± 0.08^b^	1.22 ± 0.47^b^	1.26 ± 0.17^b^

Data are mean ± s.e.m. Within columns, values followed by different superscript letters differ significantly (*P* < 0.05, Mann–Whitney Rank Sum Test).

Transcriptional analysis showed no differences in the expression of enzymes involved in anaerobic glycolysis (*GAPDH* and *LDHA*), pentose phosphate pathway (*G6PD* and *SIRT2*) and Kreb’s cycle (*CS*) between D15 embryos cultured in PHD vs N2B27 media. *IFNT2* transcript was present in both conditions, while it was more abundant in embryos cultured in PHD medium, suggesting that it may induce trophectoderm proliferation to a greater extent than N2B27 (Fig. 4D).

### Post-hatching development without agarose substrate

**Figure 5:**
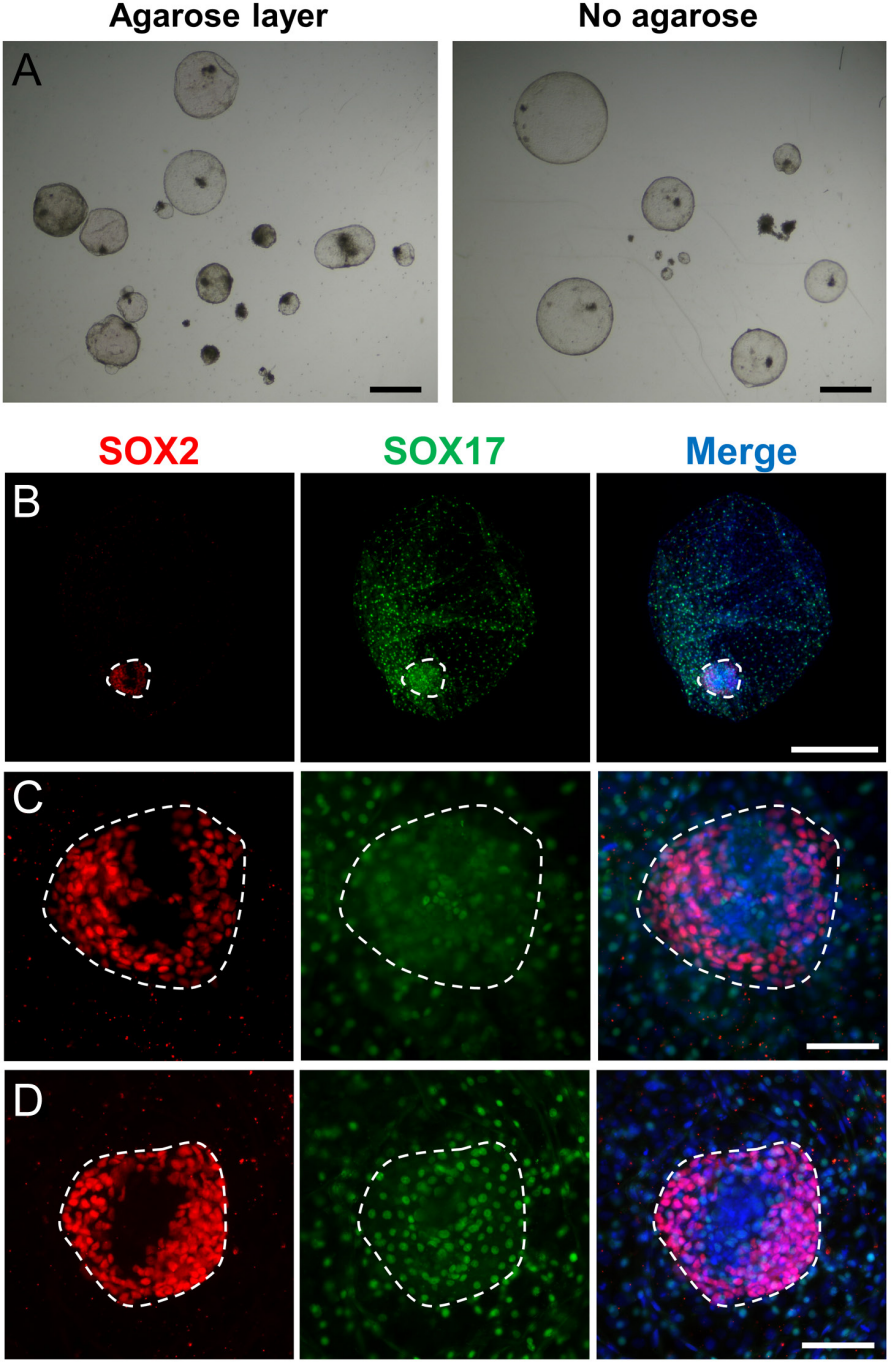
Epiblast development in N2B27 medium. (A) Representative brightfield stereomicroscopic images of D15 embryos developed in N2B27 medium in agarose-coated or agarose-free wells. (B) Epiblast and hypoblast development of a D15 representative embryo cultured in N2B27 in agarose-free well. (C) Magnification of the compact structure resembling an embryonic disc in B. (D) Representative embryonic disc-like structure from a D13 embryo cultured in N2B27 in agarose-free well. Immunofluorescence staining for SOX2 and SOX17; nuclei were counterstained with DAPI (merge). Scale bars = 1 mm for A, 500 µm for B; 100 µm for magnification in C and D.

Finally, we sought to determine if agarose substrate was necessary for bovine embryo development after blastocyst hatching. With this aim, D9 embryos were cultured in N2B27 medium until D11, measured and randomly allocated to agarose-coated or agarose-free wells with N2B27 medium. Survival rates for D11 embryos were mostly determined by embryo diameter, being similar in both groups. Only 2 out of 15 (~14%) and 5 out of 26 (~19%) D11 embryos smaller than 0.5 mm survived in agarose-coated and agarose-free wells, respectively. However, 11 out of 17 (~65%) and 16 out of 21 (~76%) D11 embryos larger than 0.5 mm survived in agarose-coated and agarose-free wells. D15 embryos showed a spherical shape and no significant differences were found in embryo survival, length, area or volume between both conditions (Fig. 5A and Table 4). All surviving embryos showed complete migration of the hypoblast in both conditions (*n* = 11 in agarose-coated and 16 in agarose-free wells, Fig. 5B). No differences were found in the number of embryos showing SOX2+ epiblast cells (6 out of 11 (~55%) in agarose-coated and 9 out of 16 (~56%) in agarose-free wells) or showing a SOX2+ compact embryonic disc-like structure (2 out of 11 (18%) in agarose-coated and 4 out of 16 (25%) in agarose-free wells). Therefore, agarose substrate is not required for bovine post-hatching development in vitro.

**Table 4:** Average length, area and volume of surviving embryos cultured in N2B27 medium on agarose-coated vs agarose-free wells at days 11 and 15 of development.

	*n*	D11 length (mm)	D11 area (mm^2^)	D11 volume (mm^3^)	D15 length (mm)	D15 area (mm^2^)	D15 volume (mm^3^)
Agarose-coated	11	0.64 ± 0.04	0.35 ± 0.04	0.16 ± 0.03	1.07 ± 0.08	0.92 ± 0.14	0.76 ± 0.17
Agarose-free	16	0.60 ± 0.05	0.32 ± 0.04	0.14 ± 0.04	1.33 ± 0.1	1.52 ± 0.26	1.57 ± 0.40

No significant differences were found (*P* > 0.05, *t*-test for D15 length and Mann–Whitney Rank Sum Test for area and volume).

## Discussion

The development of an in vitro culture system to attain bovine embryo development beyond the blastocyst stage is critical to study conceptus elongation without the need of experimental animals. Unfortunately, current methods achieve limited success, being the post-hatching development (PHD) system, described ago, the most successful so far (Brandao *et al.* 2004, Vajta *et al.* 2004). Molecular characterization of the development of embryonic lineages under the PHD system revealed that, while trophectoderm proliferated robustly, hypoblast migration was compromised and epiblast development was abolished. An initial step for system optimization was to determine the optimal culture substrate, as PHD system employed agarose tunnels. The potentially beneficial effect of agarose tunnels could be mediated by the physical constriction or by the agarose itself. Our results show that neither agarose nor physical constriction are required for embryo development, as survival rates were similar for embryos cultured inside agarose tunnels, over agarose layer or without agarose coating. Besides, although culture in PHD medium inside agarose tunnels shaped the embryo in to a cylindrical shape, embryonic growth was restricted, as embryo area and volume were significantly larger in embryos developed free-floating over an agarose layer compared to those physically restricted by agarose tunnels.

Although PHD medium did not support epiblast development and compromised hypoblast migration, trophoblast cells proliferated rapidly. Trophoblast cells developed in both PHD and N2B27 media were seemingly functional, as they expressed the trophectoderm marker CDX2 (Berg *et al.* 2011) and *IFNT2*, the major pregnancy recognition signal in ruminants (Imakawa *et al.* 1987, Helmer *et al.* 1989). Given that trophoblast growth is mainly responsible for embryonic growth, the larger embryonic size and higher transcript abundance of *IFNT2* in embryos cultured in PHD medium vs N2B27 medium suggest that PHD medium may promote trophoblast proliferation at the expense of hypoblast and epiblast proliferation. Trophectoderm culture requirements seem to be relatively less restrictive than those of other lineages, as primary bovine trophectoderm cell cultures have been established using other relatively simple media also supplemented with 10% serum (Talbot *et al.* 2000, Ramos-Ibeas *et al.* 2014). This situation contrasts with the higher demands of epiblast cells, which have been proven difficult to capture in vitro in farm animals (Ramos-Ibeas *et al.* 2018). Irrespective of which medium and substrate was used, in vitro post-hatching embryo development was largely determined by initial blastocyst size: most embryos smaller than 0.5 mm diameter on day 11 were unable to survive up to day 15 in vitro. This finding agrees with previous observations on PHD system (Brandao *et al.* 2004) and highlights the importance of pre-hatching embryo development for subsequent conceptus elongation and pregnancy success (O’Hara *et al.* 2014).

In contrast to PHD medium, N2B27 allows both complete hypoblast migration and epiblast development. Hypoblast proliferation, previously reported in the PHD system based on conventional histology and electron microscopy (Brandao *et al.* 2004), was confirmed by immunostaining for SOX17, an hypoblast marker (Canizo *et al.* 2019). However, irrespective of the substrate used, hypoblast cells did not cover the entire surface of the embryos developed in PHD medium, while all embryos developed in N2B27 medium showed complete hypoblast migration. Similarly, although we observed a compact mass of epiblast-derived cells in D15 PHD embryos, as previously reported (Brandao *et al.* 2004, Vajta *et al.* 2004), these cells did not express the epiblast marker SOX2 (Khan *et al.* 2012). The lack of epiblast markers evidences a degeneration of this lineage during PHD culture. In agreement with our results, other authors have also reported the abnormal appearance of these cell masses compared to in vivo developed embryonic discs (Machado *et al.* 2013) and abundant signs of degeneration in the form of apoptosis or necrosis evidenced by transmission electron microscopy in the PHD system (Brandao *et al.* 2004). Conversely, N2B27 medium supported epiblast survival in ~55–60% of the embryos, and compact masses of SOX2+ epiblast cells resembling embryonic discs were observed in ~18–25% of the embryos. The compact embryonic disc-like structures are similar in shape and size to an embryonic disc, but a SOX2-negative area was observed at D15 (Figs 4C and 5C). This SOX2-negative area is already present on D13, starting from the centre of the embryonic disc-like structure (Fig. 5D), suggesting that embryo requirements are still not fully fulfilled in vitro. Although further research is required to mimic maternal conditions, to our knowledge, an in vitro system achieving complete hypoblast migration and the formation of embryonic disc-like structures was not available for any ungulate or livestock species.

The requirement of a relatively high concentration of glucose in post-hatching development media (5 mg/mL in PHD and 3.6 mg/mL in N2B27, Supplementary Table 2), absent in the pre-hatching embryo culture media (0 mg/mL, Holm *et al.* 1999), coincides with an increase in glucose concentration in uterine fluid and an upregulation of different glucose transporters in the endometrium occurring during embryo elongation in ruminants (Forde *et al.* 2009, Gao *et al.* 2009*a*,*b*, Satterfield *et al.* 2009). High glucose requirement suggests a metabolic swift occurring after blastocyst hatching (Ramos-Ibeas *et al.* 2019). Our gene expression analysis of key rate-limiting enzymes revealed an increase in the expression of the anaerobic glycolysis enzymes *GAPDH* and *LDHA* after culture in PHD medium that was concomitant to a decrease of *CS*, the rate-limiting enzyme for the citric acid cycle (Seedorf *et al.* 1986), only significant when embryos were cultured free-floating. The high glucose requirement of the embryos seems to be fulfilled by both N2B27 and PHD media, since embryos developed in both media exhibited similar transcript abundance of genes encoding metabolic enzymes. The metabolic switch from oxidative phosphorylation (coupled to citric acid cycle) to anaerobic glycolysis, known as the Warburg effect, has been suggested to play a role on embryo development, arguing that, although energy production is less efficient by anaerobic glycolysis, this pathway produces metabolites that may be critical for embryo development and it may reduce the production of reactive oxygen species (ROS) (Bermejo-Alvarez *et al.* 2010*a*, Krisher & Prather 2012). In this sense, the increased expression of *CS* in embryos cultured inside tunnels compared to those free-floating may entail an increased production of ROS (Zorov *et al.* 2014). In the same line, an increase in *G6PD* and its positive regulator *SIRT2* (Wang *et al.* 2014, Lamas-Toranzo *et al.* 2018) was observed in embryos cultured in tunnel compared with those cultured over agarose layer. Glucose-6-phosphate dehydrogenase (G6PD) is the rate-limiting enzyme of the pentose phosphate pathway, a pathway producing the reducing agent NADPH, and thereby its upregulation on embryos cultured inside tunnel may respond to a higher demand for reducing ROS.

In conclusion, the three key developmental processes occurring during early embryonic elongation (trophoblast proliferation, complete hypoblast proliferation and migration, and epiblast development into an embryonic disc-like structure) can be recapitulated in an in vitro system based on N2B27 medium and not requiring agarose tunnel or layer. This system will help to understand the role of specific genes on these developmental processes and could serve as a proxy to determine embryo quality following different in vitro production methodologies.

## Supplementary Material

Supplementary Table 1. Details of primers used for qPCR.

Supplementary Table 2

Supplementary Figure 1. Superovulation protocol employed to obtain in vivo derived embryos

Supplementary Figure 2. Details for specific methods used. A) Preparation of agarose tunnels using opposing combs prepared with glass capillaries. Once the gel is prepared it needs to be washed prior to its use for embryo culture. B) Picture of the system used to take tridimensional microscopy images. PBS drops retained by circles made by PAP pen on a coverslip are covered by an incubation chamber, preventing embryo crushing.

Supplementary Figure 3. Immunofluorescence detection of hypoblast cells underneath the trophoblast layer. A) Immunofluorescence staining for SOX17 and CDX2 of a D15 embryo cultured in agarose layer, z-section in confocal microscopy. B) Magnification of the region indicated in A. Scale bars = 300 µm for A and 50 µm for B.

## Declaration of interest

The authors declare that there is no conflict of interest that could be perceived as prejudicing the impartiality of the research reported.

## Funding

This work has been funded by the projects StG-757886-ELONGAN from the European Research Councilhttp://dx.doi.org/10.13039/501100000781 and AGL2017-84908-R from the Spanish Ministry of Economy and Competitiveness (MINECO) to P B A. P R I was funded by a Talent Attraction Fellowship from Madrid Region Government (2017-T2/BIO5182) and is currently funded by a Ramón y Cajal Contract from MINECO (RYC2018-025666-I). I L T was funded by a FPI fellowship by MINECO (BES-2015-072774). A M M is funded by an Industrial Doctorate Fellowship (IND2017/BIO7748) from Madrid Region Government.

## Author contribution statement

All authors contributed to embryo production. P R I performed post-hatching embryo culture and molecular analyses. P R I and P B A designed experiments and wrote the manuscript.
