# Supplementary material for: Embryonic disc formation following post-hatching bovine embryo development in vitro
Source: Reproduction. 2020 Jul 17;160(4):579–89. doi: 10.1530/REP-20-0243 (PMC7497357; doi:10.1530/REP-20-0243)
Supplement: Supplementary Table 1. Details of primers used for qPCR. [file supplementary_table_1.pdf]

Supplementary Table 1. Details of primers used for qPCR.

Gene	Primer sequence (5'→3')	Fragment size (bp)	GeneBank accession
H2AFZ	F: AGGACGACTAGCCATGGACGTGTG<br>R: CCACCACCAGCAATTGTAGCCTTG	209	NM_174809.2
IFNT2	F: GCTATCTCTGTGCTCCATGAGATG<br>R: AGTGAGTTCAGATCTCCACCCATC	359	NM_001015511.3
LDHA	F: TTCTTAAGGAAGAACATGTC<br>R: TTCACGTTACGCTGGACCAA	310	NM_174099.2
GAPDH	F: ACCCAGAAGACTGTGGATGG<br>R: ACGCCTGCTTCACCACCTTC	247	NM_001034034.2
CS	F: ATCCTCCTAGAGCAGGGCAA<br>R: TGTGCTCATGGACTTGGGTC	204	NM_001044721.1
G6PD	F: CGCTGGGACGGGGTGCCCTTCATC<br>R: CGCCAGGCCTCCCGCAGTTCATCA	347	NM_001244135.2
SIRT2	F: GCCAGACTGACCCTTTCCTC<br>R: GGTGGTGGAGAATTCCTGGG	253	NM_001113531.1
