# Supplementary figures and images for: Embryonic disc formation following post-hatching bovine embryo development in vitro
Source: Reproduction. 2020 Jul 17;160(4):579–89. doi: 10.1530/REP-20-0243 (PMC7497357; doi:10.1530/REP-20-0243)

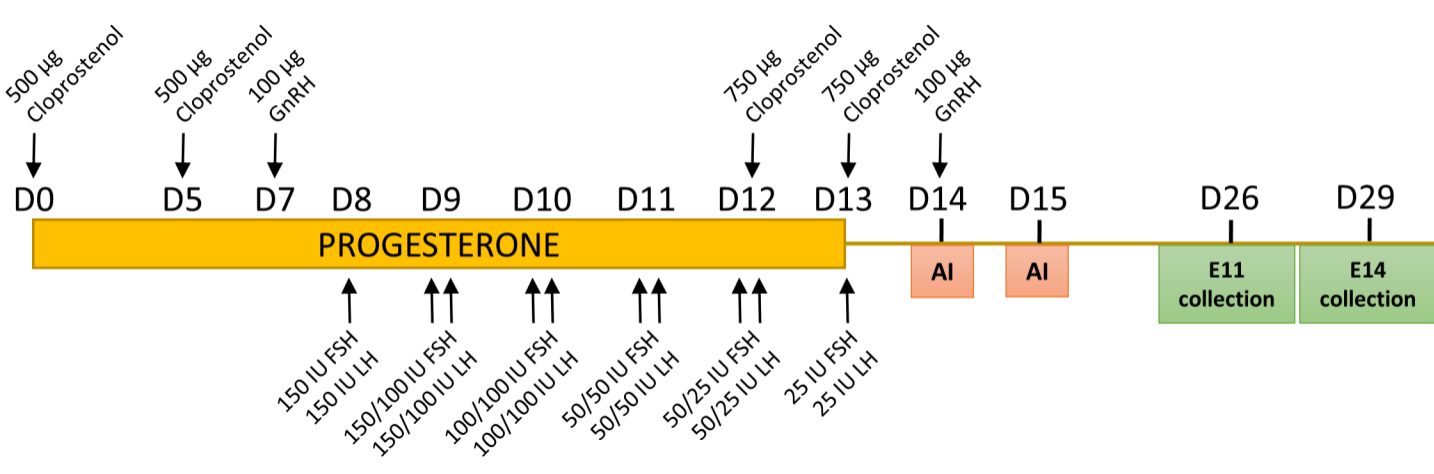

Supplement: Supplementary Figure 1. Superovulation protocol employed to obtain in vivo derived embryos [file supplementary_figure_1.pdf]

**A**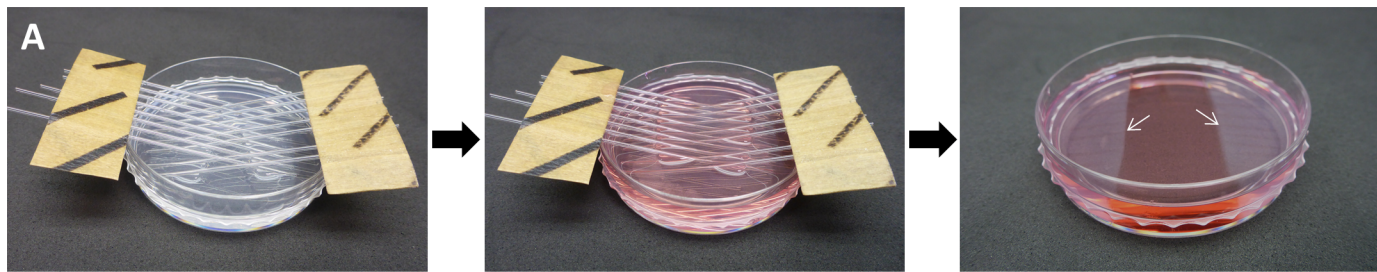**B**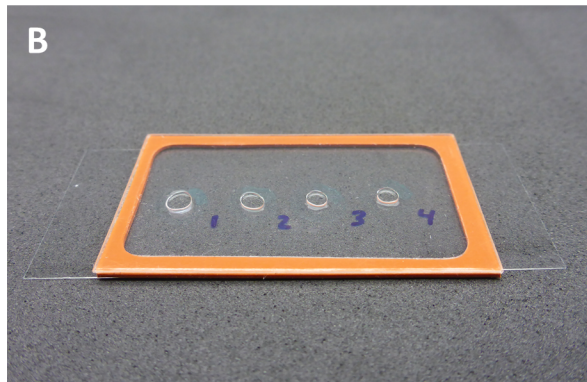

Supplement: Supplementary Figure 2. Details for specific methods used. A) Preparation of agarose tunnels using opposing combs prepared with glass capillaries. Once the gel is prepared it needs to be washed prior to its use for embryo culture. B) Picture of the system used to take tridimensional microscopy image [file supplementary_figure_2.pdf]

SOX17

CDX2

Merge

A

B

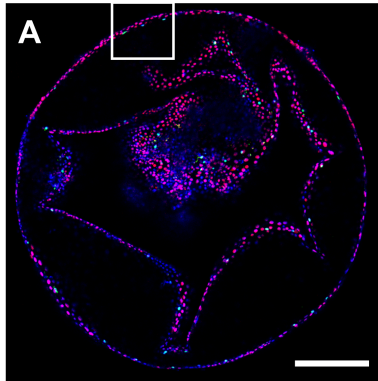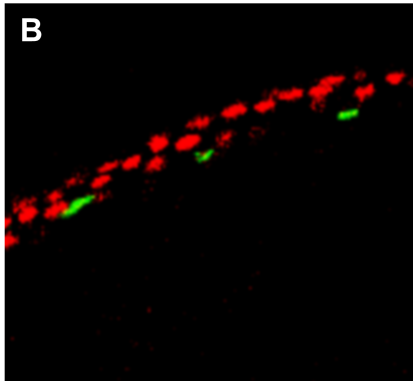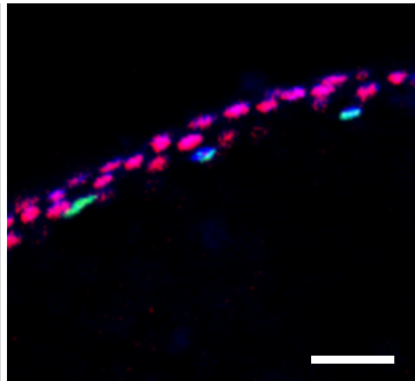

Supplement: Supplementary Figure 3. Immunofluorescence detection of hypoblast cells underneath the trophoblast layer. A) Immunofluorescence staining for SOX17 and CDX2 of a D15 embryo cultured in agarose layer, z-section in confocal microscopy. B) Magnification of the region indicated in A. Scale bars = 300 µm  [file supplementary_figure_3.pdf]
